# Extracts of Conium

**Published:** 1846-10

**Authors:** 


*Extracts of Conium.*—We have taken occasion heretofore to adduce a variety of discrepant observations with reference to the effects of the Extract of Conium, and have expressed an opinion that the great diversity of its results in different hands, is due to variations in the virtues of the articles used. Most of our American extracts are worthless, and the only good articles which we have met with have been the English extracts. We observe it mentioned, however, incidentally in an article in the Dublin Quarterly Journal of Medical Science, that "it is now well known that the officinal extracts of the London and Dublin Pharmacopœias are almost, if not completely inert." The writer adds that he has found three grains of the extract prepared from the *Succus* or expressed juice, produce symptoms of poisoning.

